# Assessment of indoor thermal comfort temperature and related behavioural adaptations: a systematic review

**DOI:** 10.1007/s11356-023-27089-9

**Published:** 2023-05-22

**Authors:** Fadly Syah Arsad, Rozita Hod, Norfazilah Ahmad, Mazni Baharom, Mohd Hasni Ja’afar

**Affiliations:** Department of Public Health Medicine, Faculty of Medicine, Universiti Kebangsaan Malaysia, 56000 Bandar Tun Razak, Kuala Lumpur Malaysia

**Keywords:** Indoor air temperature, Thermal comfort, Behavioural adaptations, Systematic review

## Abstract

Thermal comfort is linked to our health, well-being, and productivity. The thermal environment is one of the main factors that influence thermal comfort and, consequently, the productivity of occupants inside buildings. Meanwhile, behavioural adaptation is well known to be the most critical contributor to the adaptive thermal comfort model. This systematic review aims to provide evidence regarding indoor thermal comfort temperature and related behavioural adaptation. Studies published between 2010 and 2022 examining indoor thermal comfort temperature and behavioural adaptations were considered. In this review, the indoor thermal comfort temperature ranges from 15.0 to 33.8 °C. The thermal comfort temperature range varied depending on several factors, such as climatic features, ventilation mode, type of buildings, and age of the study population. Elderly and younger children have distinctive thermal acceptability. Clothing adjustment, fan usage, AC usage, and open window were the most common adaptive behaviour performed. Evidence shows that behavioural adaptations were also influenced by climatic features, ventilation mode, type of buildings, and age of the study population. Building designs should incorporate all factors that affect the thermal comfort of the occupants. Awareness of practical behavioural adaptations is crucial to ensure occupants’ optimal thermal comfort.

**Supplementary Information:**

The online version contains supplementary material available at 10.1007/s11356-023-27089-9.

## Introduction

Thermal comfort is the condition of the mind that expresses satisfaction with the thermal environment (ASHRAE 1992; Hensen 1991). Thermal sensations are different among people, even in the same environment. Several factors influence the thermal sensation, such as air temperature, air velocity, relative humidity, mean radiant temperature, clothing insulation, and activity level (Charles 2003). The thermal indices temperature-humidity index (THI), wet bulb globe temperature (WBGT), physiological effective temperature (PET), and universal thermal climate index (UTCI) were used to evaluate thermal comfort (Abdel-Ghany et al. 2013).

The steady-state-heat-balance theory and the adaptive model are the two main models in thermal comfort knowledge (Shooshtarian et al. 2020). The adaptive model’s concept depended on the personal ability to adapt to changes in their thermal environment in such a manner as to restore their comfort (Mishra and Ramgopal 2013).

There are three levels of adaptive ability: physiological, behavioural, and psychological (Schweiker et al. 2012). Behavioural adaptation is well known to be the most critical contributor to the adaptive thermal comfort model (Elnabawi and Hamza 2020; Rijal et al. 2019a). Examples of behavioural adaptation to regulate indoor thermal environments include window opening, clothing insulation, and fan or air-conditioner usage (Mishra and Ramgopal 2013; Yau and Chew 2012).

Thermal comfort standards are critical to building sustainability. High temperatures inside the buildings may provide thermal discomfort sensation and sometimes health problems such as heat stress (Abdel-Ghany et al. 2013; Ormandy and Ezratty 2016). Moreover, heat stress may lead to more severe health problems, especially in vulnerable groups such as the elderly (Lundgren Kownacki et al. 2019). However, adverse health outcomes are commonly avoidable through simple adaptive behaviour. Thus, understanding the thermal comfort indicator and behavioural adaptation to regulate indoor air temperature is necessary. In addition, thermal comfort is essential in maintaining a healthy and productive workplace.

Several studies have been conducted to measure indoor thermal comfort and identify the behavioural adaptations of the occupants of the building (Khalid et al. 2019; Kumar and Singh 2019; Rijal et al. 2019b; Shrestha et al. 2021; Zaki et al. 2017; Zheng et al. 2022). However, the findings vary depending on the study population, climate features, types of buildings, and ventilation modes.

To the best of our knowledge, there does not exist any systematic review that provides an overview of both indoor thermal comfort temperature range and related behavioural adaptations of the occupants in one study. Thus, this systematic review aims to provide evidence regarding the indoor thermal comfort temperature range in different settings. Furthermore, the findings from the review can be used to understand the adaptive behavioural preference of the study population in achieving thermal comfort.

## Methodology

The PRISMA (Preferred Reporting Items for Systematic Reviews and Meta-Analyses) review methodology, which was specifically intended for systematic reviews and meta-analyses, was used to guide this research (Page et al. 2021).

### Inclusion criteria

Included were research-based articles (field study), peer-reviewed, human participants, study findings comprising indoor thermal comfort temperature and behavioural adaptations, and English publications from 2010 to 2022.

### Study selection and data search

Related articles were identified by searching the Scopus, Web of Science, EBSCOhost, and PubMed databases. The search string was created and generated using Boolean operators and keyword search (Table 1). Following removing of duplicates, two reviewers independently examined the titles and abstracts of all identified studies to identify those that met the selection criteria.

**Table 1:** Keywords search used in the screening process

Database	Search string
Scopus	TITLE-ABS-KEY ((thermal comfort OR thermal discomfort) AND (adaptive behaviour OR adaptation OR behaviour OR practice) AND (indoor temperature OR room temperature))
Web of Science	TS = ((“thermal comfort” OR “thermal discomfort”) AND (“adaptive behaviour” OR “adaptation” OR “behaviour” OR “practice”) AND (“indoor temperature” OR “room temperature”))
PubMed	((“thermal comfort” OR “thermal discomfort”) AND (“adaptive behaviour” OR “adaptation” OR “behaviour” OR “practice”) AND (“indoor temperature” OR “room temperature”))

### Quality assessment

The quality of the selected articles was evaluated using the Navigation Guide Systematic Review framework (Woodruff and Sutton 2014) (Supplementary File. Table 1). Any disagreement was resolved with the third reviewer.

### Data extraction and synthesis

Following the initial search, we created a standardised form to extract the following data: (1) location, (2) climate and season of field study, (3) type of buildings, (4) ventilation type, (5) study population, (6) indoor comfort temperature range, and (7) adaptive behaviour.

This systematic review used a narrative synthesis encompassing quantitative and qualitative data analysis. To generate relevant themes, we used Braun and Clarke’s six-phase framework consisting of data familiarisation, code generation, theme search, theme review, theme definition, and write-up (Clarke and Braun 2013). The indoor thermal comfort temperature range and behavioural adaptations were then described separately. Finally, the quantitative and qualitative findings of the selected articles were merged using a narrative approach for the overall results.

## Result

### Basic description

**Fig. 1:**
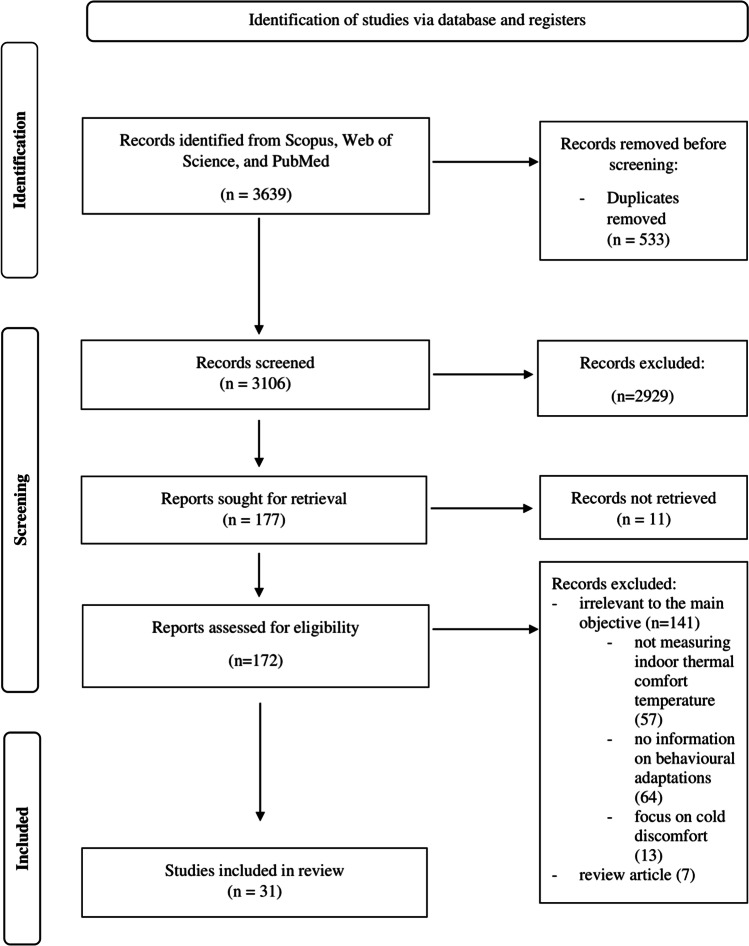
The PRISMA flow diagram

A total of 31 articles were selected based on the pre-determined inclusion criteria and analysed to identify the indoor thermal comfort temperature and the associated behavioural adaptations (Fig. 1). The included articles represented several continents in the world: Asia, 27 articles; Europe, two articles; and Oceania, two articles. In addition, the articles spanned lower- and upper-middle-income countries and high-income countries. The sample size of the studies ranged from 16 to 11,524. The studies were conducted in educational buildings (*n* = 17), residential buildings (*n* = 11), healthcare buildings (*n* = 1), and commercial/factory buildings (*n* = 3). The age of the study population ranged from 4 to 80 years old.

Table 2 summarises the characteristics and main findings of the articles included in this systematic review.

**Table 2:** Characteristics and main findings of the selected articles (*n* = 31)

No	Author	Country	Climate	Season of field study	Type of buildings	Ventilation type	Study population (age and sample size)	Comfort temperature range (°C)	Behavioural adaptations
1	Yao et al. 2010	China (Chongqing)	Subtropical	All season	University building	NV + AC	University students Age: 16–40 years old (*n* = 3621)	16.0–30.0	-Use fan -Cloth adjustment -Drink beverages
2	Teli et al. 2012	UK (Southampton)	Temperate	Spring	School building	NV	Students Age: 7–11 years old (*n* = 1300)	20.0–24.0	-Cloth adjustment
3	Pellegrino et al. 2012	India (Calcutta)	Tropical	Summer	University building	NV	University students (*n* = 100)	24.9–32.5	-Use fan
4	Liang et al. 2012	Taiwan (Taichung)	Subtropical	Autumn	Primary and high school building	NV	Students (*n* = 3754)	22.4–29.2	-Cloth adjustment
5	Rijal 2014	Japan (Kanto)	Temperate	Hot and humid	Japanese houses	NV + AC	Adult (*n* = 52)	23.0–30.0	-Use AC -Open window -Use fan
6	Baruah et al. 2014	India	Subtropical	Winter and summer	University	NV	Students (*n* = 228)	22.0–23.5 (winter) 27.3–30.7 (summer)	-Use fan -Open window -Cloth adjustment
7	Yun et al. 2014	Korea (Seoul)	Subtropical	Summer	Kindergarten	NV	Children Age: 4–6 years old (*n* = 119)	23.0–26.0	-Cloth adjustment
8	De Dear et al. 2015	Australia	Subtropical	Summer	Primary and high school	MM	Student Age: 10–18 years old (*n* = 2850)	19.5–26.6	-Use AC
9	Singh 2016	India (Chandigarh and Roorkee)	Tropical monsoon and temperate	All season	Multi-stored apartments	NV	Age: 8–80 years old (*n* = 82)	22.5–30.6	-Open window (less used during extreme summer) -Use fan -Use AC -Use blinds (daytime) -Cloth adjustment -Drink beverages -Change posture
10	Damiati et al. 2016	Malaysia Indonesia Japan Singapore	Tropical and subtropical	All season	University offices and commercial buildings	MM	Building occupants (*n* = 130)	24.5–30.0	-Use AC -Use fan -Open window and door -Drink beverages -Cloth adjustment
11	Wang et al. 2017	China (Harbin)	Subtropical	Autumn winter spring	University building	MM	University students (*n* = 30)	16.0–22.4	-Cloth adjustment
12	Liu et al. 2017b	China (Weinan and Wuwei)	Subtropical	Winter	Primary and secondary school buildings	MM	Students Age: 13–15 years old (*n* = 763)	15.0–20.0	-Cloth adjustment
13	Haddad et al. 2017	Iran (Shiraz)	Desert	All season	Primary school buildings	NV	Age: 10–12 years old (*n* = 1605)	22.0–25.0	-Cloth adjustment
14	Zaki et al. 2017	Malaysia (Shah Alam and Kuala Lumpur) Japan (Fukuoka)	Tropical and subtropical climate	Hot and humid and summer season	University buildings	MM	Students Age: 20–23 years old (*n* = 1415)	24.0–28.0	-Use AC -Drink beverages
15	Liu et al. 2017a	China	Subtropical	All season	Residential buildings	MM	Age: 20–60 years old (*n* = 11,524)	21.02–24.25	-Cloth adjustment -Use fan -Open window
16	Singh et al. 2018	India (Rajasthan)	Tropical	Summer and winter	University building	NV	University students (*n* = 900)	21.8–32.1	-Open window -Use fan
17	Khalid et al. 2019	Malaysia (Kuala Lumpur)	Tropical	Hot and humid season	Hospital buildings	AC	Mean age: 36–38 years old (*n* = 389)	22.0–28.0	-Cloth adjustment
18	Hossain et al. 2019	Bangladesh (Dhaka and Chittagong)	Tropical	Hot-dry and cold-dry season	Factory buildings	NV	Workers (*n* = 908)	22.3–31.0	-Use fan -Open windows -Reduce activity level -Tying up hair -Cloth adjustment -Change posture
19	Kumar and Singh 2019	India (Jalandhar, Jaipur)	Tropical monsoon and temperate	All season	Hostel buildings	NV	Students Age: 17–30 years old (*n* = 827)	25.8–33.8	-Cloth adjustment -Open window -Use fan
20	Rijal et al. 2019a	Japan (Kanto)	Subtropical	All season	Domestic dwellings	MM	Residents (*n* = 244)	17.0–30.0	-Open window -Use fan -Cloth adjustment
21	Kim et al. 2019	Australia (Sydney)	Subtropical	Summer and winter	University buildings	MM	University staff (*n* = 31)	23.0–25.0	-Cloth adjustment -Drink beverages -Use fan -Open window -Use AC
22	Wu et al. 2019	China (Chongqing)	Subtropical	Summer	Residential buildings and elderly nursing homes	NV	Elderly (*n* = 8) Adult (*n* = 16)	26.0–32.5	-Open window -Use fan -Use AC
23	Korsavi and Montazami 2020	UK (Coventry)	Oceanic	All season	School buildings	NV	School children Age: 9–11 years old (*n* = 805)	20.2–20.9	-Cloth adjustment -Drink beverages -Use fan -Open window
24	Malik et al. 2020	India (Mumbai)	Tropical monsoon and temperate	Monsoon, winter and summer	Residential buildings	NV	Residents Age: 18–65 years old (*n* = 705)	26.0–32.0	-Open window -Open door -Use fan -Use AC
25	Budiawan and Tsuzuki 2021	Japan (Toyohashi)	Subtropical	Summer and winter	University dormitory and residential buildings	MM	University students Mean age: 29 years old (*n* = 18)	23.5–28.1	-Cloth adjustment -Use AC
26	Shrestha et al. 2021	Nepal (Dhading, Kathmandu, and Nuwakot)	Temperate	Middle autumn	School buildings	NV	Students Age: 12–18 years old (*n* = 818)	25.0–29.0	-Cloth adjustment -Change posture -Reduce activity levels -Drink beverages -Use AC
27	Draganova et al. 2021	Japan (Toyohashi)	Subtropical	Summer	Dormitory buildings	MM	University students Age: 19–31 years old (*n* = 18)	25.0–29.0	-Use AC
28	Tsuzuki et al. 2021	Japan (Nagoya)	Subtropical	All season	Elder care facilities	MM	Elderly resident (*n* = 16)	19.0–30.0	-Cloth adjustment -Use fan -Open window
29	Zaki et al. 2021	Malaysia	Tropical	Hot and humid	2-storey residential buildings	AC	Resident Age: 23–35 years old (*n* = 20)	24.7–25.2	-Use AC -Cloth adjustment (wear less clothing)
30	Sun et al. 2022	China (Xi’an City)	Subtropical	All season	University classrooms	MM	University students Age: 16–21 years old (*n* = 578)	18.1–19.2	-Open window -Cloth adjustment -Open door -Drink beverages -Use fan -Use curtains -Use AC
31	Zheng et al. 2022	China (Guangzhou)	Subtropical	Summer	Office buildings	AC	Staffs Mean age: 23.5 years old (*n* = 34)	25.5–28.4	-Cloth adjustment -Use fan

Abbreviations: *AC*, air-conditioner; *MM*, mixed-mode; *NV*, natural ventilation; *NV* + *AC*, combination NV and AC

### Indoor thermal comfort temperature

The indoor thermal comfort temperature range is described according to the climate/season of study, ventilation mode, type of buildings, and age of the study population characteristics.

#### Tropical climate/season of study characteristic

The indoor thermal comfort temperature from the subtropical countries ranged from 15.0 to 32.5 °C, the widest range compared to other countries from different climates. Meanwhile, indoor thermal temperature comfort in tropical countries ranges from 22.0 to 33.8 °C. Studies from temperate countries showed a range from 20.0 to 30.0 °C. One study from each oceanic and desert climate country reported indoor thermal comfort temperature range of 20.2–20.9 °C and 22.0–25.0 °C respectively.

#### Ventilation modes

Mixed mode (MM) ventilation studies reported the widest indoor thermal comfort temperature range (15.0–30.0 °C). Meanwhile, the highest indoor thermal comfort temperature was reported from natural ventilation (NV) mode (20.0–33.8 °C). Studies on air-conditioner (AC) type of ventilation showed the narrowest range of indoor thermal comfort temperature (22.0–28.4 °C). Studies on the NV + AC mode combination ranged from 16.0 to 30.0 °C.

#### Type of buildings

Studies on educational buildings reported the broadest range of indoor thermal comfort temperatures (15.0–32.5 °C). The narrowest range came from the healthcare building study (22.0–28.0 °C). Residential and commercial buildings reported indoor thermal comfort temperature ranges of 17.0–33.8 °C and 22.3–31.0 °C, respectively.

#### Age of study population

Most articles reported findings on school and university students with indoor thermal comfort temperatures ranging from 15.0 to 33.8 °C. One study focused on kindergarten showed the narrowest range of indoor thermal comfort (23.0–26.0 °C). Meanwhile, a study on the elderly showed the broadest indoor thermal comfort temperature range (19.0–30.0 °C).

### Adaptive behaviour

The adaptive behaviour is described according to the ventilation mode, type of buildings, and age of the study population characteristics.

#### Ventilation mode

In the NV mode, cloth adjustment was the highest adaptive behaviour with ten articles, followed by fan usage with nine articles. The third highest was the opening window (eight articles). The fourth highest adaptive behaviour was AC usage. Meanwhile, drinking beverages and posture change were the fifth most frequent adaptive behaviour, with three articles respectively. Reducing activity levels was the sixth highest adaptive behaviour, with two articles. Blind/curtain usage, opening door, and tying up hair were the least adaptive behaviour reported with one article each.

For MM mode, cloth adjustment was the highest adaptive behaviour with nine articles. AC usage was the second-highest adaptive behaviour (seven articles). Fan usage and open window were the third highest, with six articles each, followed by drink beverages. Two articles reported open windows as one of the adaptive behaviour preferred by the occupants. Blind/curtain usage was the least adaptive behaviour reported for MM mode.

Similar to NV and MM modes, cloth adjustment was the famous adaptive behaviour performed in AC mode (three articles). Other than that, the occupant preferred to use AC and fan (one article each). For NV + AC mode, fan usage was the highest adaptive behaviour reported in the two articles, then, followed by cloth adjustment, drink beverages, AC usage, and open window with one article each.

#### Type of buildings

In the educational buildings, the highest reported adaptive behaviour from the occupants was cloth adjustment with 12 articles, followed by fan usage (seven articles) and drink beverages with six articles. AC usage and open window were the fourth highest adaptive behaviour (five articles each). The least adaptive behaviour reported in this type of building was posture change and reduced activity level with one article each.

In the residential buildings, the highest adaptive behaviour performed by the occupants was opening window and fan usage, with seven articles, respectively. AC usage and cloth adjustment were the second-highest reported adaptive behaviour, with six articles each. The least adaptive behaviour practised was opening the door (one article).

Meanwhile, in commercial/factory buildings, the most common adaptive behaviour was fan usage and cloth adjustment (three articles each). The practice of opening the window was the second highest with two articles. The rest were AC usage, open door, drinking beverages, reduced activity level, tying up hair, and posture change (one article each). In the healthcare building, cloth adjustment was the only adaptive behaviour reported.

#### Age of study population

In this review, the elderly preferred fan usage, open window, and cloth adjustment as their adaptive behaviour for thermal comfort. Meanwhile, kindergarten children choose cloth adjustment for adaptive behaviour. For the primary and high school students, cloth adjustment was the highest adaptive behaviour reported in six articles. AC usage and drink beverages were the second highest with two articles. The least practised by primary and high school students were posture change and reduced activity level. For university students, fan usage was the highest reported adaptive behaviour (seven articles). Cloth adjustment was the second, with six articles. AC usage and open window were the third highest with five articles. Drinking beverages was the fourth (four articles), followed by the open door and curtain/blind usage with one article each.

Table 3 depicts the summary of behavioural adaptations practised by the study population.

**Table 3:** Behavioural adaptations practices

Author	Use AC	Use fan	Open window	Open door	Drink beverages	Cloth adjustment	Use curtain/blind	Posture change	Reduce activity level	Tying up hair
Rijal 2014	√	√	√							
Singh 2016	√	√	√		√	√	√	√		
Damiati et al. 2016	√	√	√	√	√	√				
Zaki et al. 2017	√				√					
Liu et al. 2017a		√	√			√				
Khalid et al. 2019						√				
Hossain et al. 2019		√	√			√		√	√	√
Kumar and Singh 2019		√	√			√				
Rijal et al. 2019a		√	√			√				
Kim et al. 2019	√	√	√		√	√				
Wu et al. 2019	√	√	√							
Korsavi and Montazami 2020		√	√		√	√				
Malik et al. 2020	√	√	√	√						
Budiawan and Tsuzuki 2021	√					√				
Shrestha et al. 2021	√				√	√		√	√	
Draganova et al. 2021	√									
Tsuzuki et al. 2021		√	√			√				
Zaki et al. 2021	√					√				
Sun et al. 2022	√	√	√	√	√	√	√			
Zheng et al. 2022		√				√				
Yao et al. 2010		√			√	√				
Teli et al. 2012						√				
Pellegrino et al. 2012		√								
Liang et al. 2012						√				
Baruah et al. 2014		√	√			√				
Yun et al. 2014						√				
De Dear et al. 2015	√									
Haddad et al. 2017						√				
Liu et al. 2017b						√				
Wang et al. 2017						√				
Singh et al. 2018		√	√							
Total	13	18	15	3	8	23	2	3	2	1

## Discussion

This review explored the literature on the critical issues of indoor thermal comfort temperature and behavioural adaptations. The findings in this review showed the range of indoor temperature for thermal comfort and the diversity of behavioural adaptations practised in this systematic review. Furthermore, the indoor thermal comfort temperature and related adaptive behaviour in this review varied depending on the climate/season characteristic, type of ventilation, type of buildings, and the age of the study population.

The widest range of thermal comfort temperature was found in the studies conducted in subtropical countries, especially countries with four or more seasons in a year. Compared to other climate characteristics, these subtropical countries will have two distinguished conditions: stronger summer and more robust winter (Cherchi et al. 2018). Thus, the population in this climate will adapt to the lower and higher temperature and explain the broadest range of thermal comfort temperatures. Meanwhile, tropical, desert, and temperate countries’ populations have a narrower range in this study. The acclimatisation factor also significantly explains the difference in the threshold of thermal comfort temperatures (Yamtraipat et al. 2005). Prolonged exposure to cold or hot temperatures will stimulate the acute and long-term physiological response to maintain the person’s thermal balance (Buckley et al. 2015; Castellani and Young 2016; Chong and Zhu 2017). Other than that, these findings support that the outdoor temperature affected indoor thermal acceptability (Yan et al. 2016).

Different type of ventilation in the building also affected the indoor thermal comfort. In this study, the thermal comfort temperature range was the narrowest compared to other modes of ventilation (MM, NV, and NV + AC). Acclimatisation factors play a significant role in explaining this finding (Chong et al. 2019; Parsons 2002; Yamtraipat et al. 2005). A building that operates with AC mode will always ensure low indoor temperature. The occupants in these buildings need to acclimatise to this temperature. The residents of traditional buildings rely on fans and traditional ventilations, which raises the indoor temperature slightly higher than in air-conditioned buildings (Hailu et al. 2021). Once again, the acclimatisation factors determine the temperature thermal comfort.

For the age of the study population, the elderly showed the widest range of thermal comfort temperature compared to the other age groups. Ageing causes physiological debility in the elderly (Boss and Seegmiller 1981; Amarya et al. 2018). Older people are more insensitive to changes in their surroundings than young people; i.e., they are unable to detect thermal stresses immediately and have a more difficult time recovering from a previous hot thermal stress (Xiong et al. 2019). However, despite this broader range of thermal comfort temperatures, this may not satisfy the long-term physical health requirements of the elderly (Fan et al. 2017). Meanwhile, younger children demonstrated a narrower indoor thermal comfort temperature range in this study. Children have lower comfort temperatures and higher sensitivity to temperature changes, especially during heating seasons (Korsavi and Montazami 2020). Thus, the building design should consider the age of the occupants to help these group achieve their optimal thermal comfort.

Cloth adaptation has become the most famous adaptation behaviour for attaining thermal comfort. Clothing acts as a barrier to thermal balance in hot environments by inhibiting evaporative and convective cooling (Davis and Bishop 2013). Since each person’s thermal need is unique and dynamic, cloth adjustments can control a person’s micro-environment between the body skin and the indoor temperature (Liu et al. 2022). Students in naturally ventilated classrooms will likely practice cloth adjustment due to limited adaptivity opportunities (Jia et al. 2021). They might not be capable of opening or closing windows or changing their activity level to adapt to specific environments according to their will (De Giuli et al. 2012).

Furthermore, this method reduces energy consumption compared to an air conditioner (Jia et al. 2021). However, it can be a challenge to the people, especially during working hours or schooling, requiring them to dress appropriately according to the dress code. A study in India reported that clothing insulation was insignificant due to socio-cultural control, attitude, and lifestyle (Indraganti 2010). In addition, the person’s desire to maintain their privacy in front of others influences their preference for wearing light clothing only inside their room (Indraganti 2010). Therefore, some of the population in this review only prefer wearing light clothing inside their room or house to achieve thermal comfort. Despite all these challenges, health awareness of the benefits of this simple and practical method should be empowered, mainly to prevent the adverse effect of high-temperature exposure to health.

Fan usage is another most common adaptative behaviour for attaining thermal comfort. It is the most practical and cost-friendly to the residents. Most buildings are equipped with either a ceiling fan or a standing fan. Like air-conditioning, the fan’s function can rapidly cool the air temperature (Ho et al. 2009). The advantage of using a fan instead was the lower greenhouse gas emissions and cost than air conditioning (Morris et al. 2021). The energy consumption is also lower than the usage of the air-conditioner. One of the limitations of this method is that it depends on the air temperature and relative humidity. In extreme temperatures, such as temperatures exceeding 35 °C, international public health organisations are not recommended to use of fans (Morris et al. 2021). Despite this drawback, the affordability and availability of this method in almost all buildings make fan usage the first choice for their behavioural adaptations.

Good ventilation is essential for thermal comfort (Costa et al. 2019). Opening a window can be an efficient way to remove hot indoor temperatures. On the other hand, good ventilation improves the thermal environment and helps remove indoor pollutants from a building (Chatzidiakou et al. 2015; Lipczynska et al. 2015). Performing this method will help improve thermal comfort and health. In this review, the only concern of the person performing this method will be the safety issue. As colder air is usually at night, opening a window to let the colder air enter their house can be problematic for some people. Safety reasons such as being afraid of burglary or wild animals entering their place will prevent the population from practising this simple method (Indraganti 2010). It is illustrated in this review, where the person prefers to open the window only during the daytime. However, the public should practice this method as the benefits outweigh the disadvantage, especially in improving thermal comfort and health.

The usage of air-conditioners becomes another typical famous adaptation behaviour for thermal comfort in this review. The advantage of using an air-conditioner is the rapid action to cool down hot and humid conditions (Lee and Tsai 2020). Furthermore, based on these articles, most respondents chose air conditioning due to the machine’s availability inside the buildings. For example, air conditioning usage in hospitals is critical for patient comfort and the productivity of healthcare workers (Khodakarami and Nasrollahi 2012; Pereira et al. 2020). Chronic patients with cardiovascular, respiratory, and many other chronic diseases inside the hospital are vulnerable to high-temperature exposure (Ebi et al. 2021; Kenny et al. 2010). According to a multi-country longitudinal study, air conditioning is an effective heat adaptation strategy associated with a lower risk of heat-related mortality (Sera et al. 2020). Thus, maintaining a colder environment inside this building is crucial for the comfort and health aspect of the population inside the building. Prolonged exposure to this lower temperature will lead to the acclimatisation of the population that prefer a lower temperature to maintain their thermal comfort (Yu et al. 2012). However, the availability of air conditioners in all buildings, especially residential ones, can be an issue, especially for the lower socioeconomic population that cannot afford them. Furthermore, using air conditioners will release more greenhouse gas emissions, leading to global warming (Al‐Ghussain 2019). Thus, the usage of this method should be used wisely.

Simple adaptive behaviours such as consuming beverages or water can improve thermal comfort. High temperatures can cause loss of body fluid that is important for thermal regulation (Osilla et al. 2022). This simple and practical method should be applied by the building’s residents, especially the elderly. The elderly was insensitive to the changing surrounding temperatures (Coccarelli et al. 2018). The population can adapt to higher indoor temperatures by consuming water or beverages and preventing more serious adverse effects like dehydration (Benelam and Wyness 2010).

Metabolic rate also plays an essential role in determining a person’s thermal comfort (Hasan et al. 2016). Higher-intensity activities will generate much heat and increase the person’s body temperature (Kenny and Mcginn 2017). Therefore, limiting the activities can help attain thermal comfort. In this review, performing light activities (~ Met 1.0) will adapt to high indoor temperatures. However, this method might not be practical, especially for workers requiring heavy-duty activities for their job scope. Postural changes also can be an effective and practical adaptive behaviour to improve thermal comfort. A change in posture can alter the effective body surface area available for heat exchange with the environment and, thus, the metabolic rate per unit body surface area (Raja and Nicol 1997). Similar to reduced activity levels, the population’s nature or work might prevent them from practising this method. Many workers, especially from developing countries, had moderate to heavy duties.

Other than that, using shade or curtain became a choice as it can block the radiation from outside to go inside the house and maintain a cooler indoor air temperature (Kim et al. 2012). However, this adaptive behaviour is only helpful during the daytime. This limitation might be why this method is not popularly found in this review.

Other least common methods practised by the study population to attain thermal comfort were opening the door and tying up hair. Opening the door to improve thermal comfort temperature was similar to opening the window. However, this method is not popular mainly due to security reasons (Indraganti 2010). Tying up hair helps in improving the heat released from the body. However, this method may only be effective and suitable for some people. Interestingly, some respondents chose not to do any adaptation behaviour to attain thermal comfort (Zaki et al. 2017). One of the possible explanations is the previously mentioned of physiological acclimatisation factor (Buckley et al. 2015).

### Limitation

This study has some limitations. This study does not include a detailed review of environmental factors, including air temperature, air velocity, humidity, and radiation, that are important in determining thermal comfort (Halawa et al. 2014; Simion et al. 2016). However, this study only focuses on indoor thermal comfort temperature as this review aims to assess the indoor thermal comfort temperature range and related adaptive behaviour. Other than that, the limited number of study healthcare buildings and studies from desert, oceanic, and temperate countries may not generalise the finding in this study on these factors. However, since this systematic review only selected articles from the predetermined inclusion criteria and underwent a systematic selection process, the included articles were sufficient to achieve the study objectives.

## Conclusion

The indoor thermal comfort temperature range and related adaptive behaviour in this study varied depending on several factors, such as climatic features, ventilation types, type of buildings, and age of the study population. All these factors were related to each other. The elderly have the broadest range of indoor thermal comfort temperatures. Meanwhile, younger children showed the opposite findings. Clothing insulation, fan usage, AC usage, and open window were the most practised by occupants to attain thermal comfort. Thus, building designs should consider the above factors for improving indoor thermal comfort environments to benefit the occupants in the long-term. Community awareness of these adaptive behaviours should be empowered, as thermal discomfort can harm health and productivity performance.

## Supplementary Information

Below is the link to the electronic supplementary material.

Supplementary file1 (DOCX 21 KB)

## Data Availability

Data is contained within the article or supplementary material.

## References

[CR1] Abdel-Ghany AM, Al-Helal IM, Shady MR (2013) Human thermal comfort and heat stress in an outdoor urban arid environment: a case study. Adv Meteorol 2013:1:7. 10.1155/2013/693541

[CR2] Al-Ghussain L (2019). Global warming: review on driving forces and mitigation. Environ Prog Sustain Energy.

[CR3] Amarya S, Singh K, Sabharwal MS (2018) Ageing Process and Physiological Changes. InTech. 10.5772/intechopen.76249

[CR4] ASHRAE (1992) ANSI/ASHRAE Standard 55-1992, thermal environmental conditions for human occupancy. American Society of Heating, Refrigerating and Air Conditioning Engineers, Inc, Atlanta

[CR5] Baruah P, Singh MK, Mahapatra S (2014) Thermal comfort in naturally ventilated classrooms. 30th International PLEA Conference: Sustainable Habitat for Developing Societies: Choosing the Way Forward-Proceedings, Ahmedabad, India. https://bit.ly/2P7AZXf.hlm. Access date: 10 May 2022

[CR6] Benelam B, Wyness L (2010) Hydration and health: a review. Nutr Bull 35(1):3–25. 10.1111/j.1467-3010.2009.01795.x

[CR7] Boss GR, Seegmiller JE (1981). Age-related physiological changes and their clinical significance. West J Med.

[CR8] Buckley LB, Ehrenberger JC, Angilletta MJ (2015). Thermoregulatory behaviour limits local adaptation of thermal niches and confers sensitivity to climate change. Funct Ecol.

[CR9] Budiawan W, Tsuzuki K (2021). Thermal comfort and sleep quality of indonesian students living in Japan during summer and winter. Buildings.

[CR10] Castellani JW, Young AJ (2016). Human physiological responses to cold exposure: acute responses and acclimatization to prolonged exposure. Auton Neurosci.

[CR11] Charles KE (2003) Fanger's thermal comfort and draught models. research report (National Research Council of Canada. Institute for Research in Construction); no. IRC-RR-162. National Research Council of Canada. 10.4224/20378865

[CR12] Chatzidiakou L, Mumovic D, Summerfield A (2015). Is Co2 a good proxy for indoor air quality in classrooms? Part 1: the interrelationships between thermal conditions, Co2 levels, ventilation rates and selected indoor pollutants. Build Serv Eng Res.

[CR13] Cherchi A, Ambrizzi T, Behera SK, Freitas ACV, Morioka Y, Zhou T (2018). The response of subtropical highs to climate change. Curr Clim Chang Rep.

[CR14] Chong D, Zhu N (2017). Human heat acclimatization in extremely hot environments: a review. Procedia Eng.

[CR15] Chong D, Zhu N, Luo W, Zhang Z (2019). Broadening human thermal comfort range based on short-term heat acclimation. Energy.

[CR16] Clarke V, Braun V (2013) Teaching thematic analysis: overcoming challenges and developing strategies for effective learning. The Psychologist 26:120–123

[CR17] Coccarelli A, Hasan HM, Carson J, Parthimos D, Nithiarasu P (2018). Influence of ageing on human body blood flow and heat transfer: a detailed computational modelling study. Int J Numer Method Biomed Eng.

[CR18] Costa ML, Freire MR, Kiperstok A (2019). Strategies for thermal comfort in university buildings-the case of the Faculty of Architecture at the Federal University of Bahia, Brazil. J Environ Manag.

[CR19] Damiati SA, Zaki SA, Rijal HB, Wonorahardjo S (2016). Field study on adaptive thermal comfort in office buildings in Malaysia, Indonesia, Singapore, and Japan during hot and humid season. Build Environ.

[CR20] Davis J-K, Bishop PA (2013). Impact of clothing on exercise in the heat. Sports Med.

[CR21] De Dear R, Kim J, Candido C, Deuble M (2015). Adaptive thermal comfort in Australian school classrooms. Build Res Inform.

[CR22] De Giuli V, Da Pos O, De Carli M (2012). Indoor environmental quality and pupil perception in Italian primary schools. Build Environ.

[CR23] Draganova VY, Yokose H, Tsuzuki K, Nabeshima Y (2021). Field study on nationality differences in adaptive thermal comfort of university students in dormitories during summer in Japan. Atmosphere.

[CR24] Ebi KL, Capon A, Berry P, Broderick C, de Dear R, Havenith G, Honda Y, Kovats RS, Ma W, Malik A, Morris NB, Nybo L, Seneviratne SI, Vanos J, Jay O (2021) Hot weather and heat extremes: health risks. Lancet (London, England) 398(10301):698–708. 10.1016/S0140-6736(21)01208-3 10.1016/S0140-6736(21)01208-3 34419205

[CR25] Elnabawi MH, Hamza N (2020) Behavioural perspectives of outdoor thermal comfort in urban areas: a critical review. Atmosphere 11(1):51. 10.3390/atmos11010051

[CR26] Fan G, Xie J, Yoshino H, Yanagi, U, Hasegawa K, Wang C, Zhang X, Liu J (2017) Investigation of indoor thermal environment in the homes with elderly people during heating season in Beijing, China. Build Environ 126. 10.1016/j.buildenv.2017.09.031

[CR27] Haddad S, Osmond P, King S (2017). Revisiting thermal comfort models in Iranian classrooms during the warm season. Build Res Inform.

[CR28] Hailu H, Gelan E, Girma Y (2021) Indoor thermal comfort analysis: a case study of modern and traditional buildings in hot-arid climatic region of Ethiopia. Urban Sci 5(3):53. 10.3390/urbansci5030053

[CR29] Halawa E, Van Hoof J, Soebarto V (2014). The impacts of the thermal radiation field on thermal comfort, energy consumption and control—a critical overview. Renew Sustain Energy Rev.

[CR30] Hasan MH, Alsaleem F, Rafaie M (2016). Sensitivity study for the PMV thermal comfort model and the use of wearable devices biometric data for metabolic rate estimation. Buil Environ.

[CR31] Hensen JLM (1991) On the thermal interaction of building structure and heating and ventilating system. [Phd Thesis 1 (Research TU/e / Graduation TU/e), Built Environment]. Technische Universiteit Eindhoven. 10.6100/IR353263

[CR32] Ho SH, Rosario L, Rahman MM (2009). Thermal comfort enhancement by using a ceiling fan. Appl Therm Eng.

[CR33] Hossain MM, Wilson R, Lau B, Ford B (2019). Thermal comfort guidelines for production spaces within multi-storey garment factories located in Bangladesh. Build Environ.

[CR34] Indraganti M (2010). Adaptive use of natural ventilation for thermal comfort in Indian apartments. Build Environ.

[CR35] Jia L-R, Han J, Chen X, Li Q-Y, Lee C-C, Fung Y-H (2021) Interaction between thermal comfort, indoor air quality and ventilation energy consumption of educational buildings: a comprehensive review. Buildings 11(12):591. 10.3390/buildings11120591

[CR36] Kenny GP, McGinn R (2017) Restoration of thermoregulation after exercise. J Appl Physiol (Bethesda, Md : 1985) 122(4):933–944. 10.1152/japplphysiol.00517.2016 10.1152/japplphysiol.00517.2016 27881668

[CR37] Kenny GP, Yardley J, Brown C, Sigal RJ, Jay O (2010) Heat stress in older individuals and patients with common chronic diseases. CMAJ : Canadian Medical Association Journal = journal de l'Association medicale canadienne 182(10):1053–1060. 10.1503/cmaj.081050 10.1503/cmaj.081050 PMC2900329 19703915

[CR38] Khalid W, Zaki SA, Rijal HB, Yakub F (2019). Investigation of comfort temperature and thermal adaptation for patients and visitors in Malaysian hospitals. Energy Build.

[CR39] Khodakarami J, Nasrollahi N (2012). Thermal comfort in hospitals–a literature review. Renew Sustain Energy Rev.

[CR40] Kim J, Tartarini F, Parkinson T, Cooper P, De Dear R (2019). Thermal comfort in a mixed-mode building: are occupants more adaptive?. Energy Build.

[CR41] Kim G, Lim HS, Lim T, Schaefer L, Kim J (2012) Comparative advantage of an exterior shading device in thermal performance for residential buildings. Energy Build 46:105-111

[CR42] Korsavi SS, Montazami A (2020). Children’s thermal comfort and adaptive behaviours; UK primary schools during non-heating and heating seasons. Energy Build.

[CR43] Kumar S, Singh MK (2019). Field investigation on occupant’s thermal comfort and preferences in naturally ventilated multi-storey hostel buildings over two seasons in India. Build Environ Prog Sustain Energy.

[CR44] Lee D, Tsai F-P (2020) Air conditioning energy saving from cloud-based artificial intelligence: case study of a split-type air conditioner. Energies 13(8):2001. 10.3390/en13082001

[CR45] Liang H-H, Lin, TP, Hwang R-L (2012) Linking occupants' thermal perception and building thermal performance in naturally ventilated school buildings. Appl Energy 94. 10.1016/j.apenergy.2012.02.004

[CR46] Lipczynska A, Kaczmarczyk J, Melikov AK (2015). Thermal environment and air quality in office with personalized ventilation combined with chilled ceiling. Build Environ.

[CR47] Liu HW, Li Y, Cheng B, Yong Yao R (2017). Seasonal variation of thermal sensations in residential buildings in the hot summer and cold winter zone of China. Energy Buil.

[CR48] Liu Y, Jiang J, Wang D, Liu J (2017). The indoor thermal environment of rural school classrooms in northwestern China. Indoor Built Environ.

[CR49] Liu JF, Worre I, Moeslund TB (2022). Clothing insulation rate and metabolic rate estimation for individual thermal comfort assessment in real life. J Sensors.

[CR50] Lundgren Kownacki K, Gao C, Kuklane K, Wierzbicka A (2019). Heat stress in indoor environments of Scandinavian urban areas: a literature review. Int J Environ Res Public Health.

[CR51] Malik J, Bardhan R, Hong T, Piette MA (2020). Contextualising adaptive comfort behaviour within low-income housing of Mumbai, India. Build Environ.

[CR52] Mishra AK, Ramgopal M (2013). Field studies on human thermal comfort—an overview. Build Environ.

[CR53] Morris NB, Chaseling GK, English T, Gruss F, Maideen MFB, Capon A, Jay O (2021) Electric fan use for cooling during hot weather: a biophysical modelling study. Lancet Planet Health 5(6):e368–e377. 10.1016/S2542-5196(21)00136-4 10.1016/S2542-5196(21)00136-4 34119011

[CR54] Ormandy D, Ezratty V (2015) Thermal discomfort and health: protecting the susceptible from excess cold and excess heat in housing. Adv Build Energy Res 10:1–15. 10.1080/17512549.2015.1014845

[CR55] Osilla EV, Marsidi JL, Sharma S (2022). Physiology, temperature regulation.

[CR56] Page MJ, McKenzie JE, Bossuyt PM, Boutron I, Hoffmann TC, Mulrow CD, Shamseer L, Tetzlaff JM, Moher D (2021) Updating guidance for reporting systematic reviews: development of the PRISMA 2020 statement. J Clin Epidemiol 134:103–112. 10.1016/j.jclinepi.2021.02.003 10.1016/j.jclinepi.2021.02.003 33577987

[CR57] Parsons KC (2002). The effects of gender, acclimation state, the opportunity to adjust clothing and physical disability on requirements for thermal comfort. Energy Build.

[CR58] Pellegrino M, Simonetti M, Fournier L (2012) A field survey in Calcutta. Architectural issues, thermal comfort and adaptive mechanisms in hot humid climates. 7th Wind. Conf., pp 12–15. https://hal.science/hal-01205783

[CR59] Pereira PFC, Broday EE, Xavier AAP (2020) Thermal comfort applied in hospital environments: a literature review. Appl Sci 10(20):7030. 10.3390/app10207030

[CR60] Raja IA, Nicol F (1997) A technique for recording and analysis of postural changes associated with thermal comfort. Appl Ergon 28(3):221–225. 10.1016/s0003-6870(96)00036-1 10.1016/s0003-6870(96)00036-1 9414361

[CR61] Rijal H, Humphreys Ma, Nicol Jf (2019). Adaptive model and the adaptive mechanisms for thermal comfort in Japanese dwellings. Energy Build.

[CR62] Rijal HB, Humphreys MA, Nicol JF (2019) Behavioural adaptation for the thermal comfort and energy saving in Japanese offices. J Inst Eng 15(2):14–25. 10.3126/jie.v15i2.27637

[CR63] Rijal HB (2014) Investigation of comfort temperature and occupant behavior in Japanese houses during the hot and humid season 4(3):437-452

[CR64] Schweiker M, Brasche S, Bischof W, Hawighorst M, Voss K, Wagner A (2012) Development and validation of a methodology to challenge the adaptive comfort model. Build Environ 49:336–347. 10.1016/J.BUILDENV.2011.08.002

[CR65] Sera F, Hashizume M, Honda Y, Lavigne E, Schwartz J, Zanobetti A, Tobias A, Iñiguez C, Vicedo-Cabrera AM, Blangiardo M, Armstrong B, Gasparrini A (2020) Air conditioning and heat-related mortality: a multi-country longitudinal study. Epidemiology (Cambridge, Mass) 31(6):779–787. 10.1097/EDE.0000000000001241 10.1097/EDE.0000000000001241 33003149

[CR66] Shooshtarian S, Lam CKC, Kenawy I (2020). Outdoor thermal comfort assessment: a review on thermal comfort research in Australia. Build Environ.

[CR67] Shrestha M, Rijal H, Kayo G, Shukuya M (2021). A field investigation on adaptive thermal comfort in school buildings in the temperate climatic region of Nepal. Build Environ.

[CR68] Simion M, Socaciu L, Unguresan P (2016). Factors which influence the thermal comfort inside of vehicles. Energy Procedia.

[CR69] Singh M, Kumar S, Ooka R, Rijal H, Gupta G, Kumar A (2018) Status of thermal comfort in naturally ventilated classrooms during the summer season in the composite climate of India. Build Environ 128:287–304. 10.1016/j.buildenv.2017.11.031

[CR70] Singh S (2016) Seasonal evaluation of adaptive use of controls in multi-storied apartments: a field study in composite climate of north India. Int J Sustain Built Environ 5(1):83–98

[CR71] Sun Y, Luo X, Ming H (2022) Analyzing the time-varying thermal perception of students in classrooms and its influencing factors from a case study in Xi’an, China. Buildings 12(1):75. 10.3390/buildings12010075

[CR72] Teli D, Jentsch MF, James PA (2012). Naturally ventilated classrooms: an assessment of existing comfort models for predicting the thermal sensation and preference of primary school children. Energy Build.

[CR73] Tsuzuki K, Sakoi T, Sakata Y (2021) Effect of seasonal ambient temperature on sleep and thermal comfort in older people living in public elderly facilities. Buildings 11(12):574. 10.3390/buildings11120574

[CR74] Wang Z, Ning H, Zhang X, Ji Y (2017). Human thermal adaptation based on university students in China’s severe cold area. Sci Technol Built Environ.

[CR75] Woodruff TJ, Sutton P (2014) The Navigation Guide systematic review methodology: a rigorous and transparent method for translating environmental health science into better health outcomes. Environ Health Perspect 122(10):1007–1014. 10.1289/ehp.1307175 10.1289/ehp.1307175 PMC4181919 24968373

[CR76] Wu Y, Liu H, Li B, Kosonen R, Deyu K, Zhou S, Yao R (2019) Thermal adaptation of the elderly during summer in a hot humid area: psychological, behavioral, and physiological responses. Energy Build 203:109450. 10.1016/j.enbuild.2019.109450

[CR77] Xiong J, Ma T, Lian Z, De Dear R (2019). Perceptual and physiological responses of elderly subjects to moderate temperatures. Build Environ.

[CR78] Yamtraipat N, Khedari J, Hirunlabh J (2005). Thermal comfort standards for air conditioned buildings in hot and humid thailand considering additional factors of acclimatization and education level. Sol Energy.

[CR79] Yan H, Yang L, Zheng W, Li D (2016). Influence of outdoor temperature on the indoor environment and thermal adaptation in Chinese residential buildings during the heating season. Energy Build.

[CR80] Yao R, Liu J, Li B (2010) Occupants’ adaptive responses and perception of thermal environment in naturally conditioned university classrooms. Appl Energy 87(3):1015–1022

[CR81] Yau Y, Chew BT (2012) A review on predicted mean vote and adaptive thermal comfort models. Build Serv Eng Res Technol 35:23–35. 10.1177/0143624412465200

[CR82] Yu J, Ouyang Q, Zhu Y, Shen H, Cao G, Cui W (2012). A comparison of the thermal adaptability of people accustomed to air-conditioned environments and naturally ventilated environments. Indoor Air.

[CR83] Yun H, Nam I, Kim J, Yang J, Lee K, Sohn J (2014) A field study of thermal comfort for kindergarten children in Korea: an assessment of existing models and preferences of children. Build Environ 75:182–189. 10.1016/j.buildenv.2014.02.003

[CR84] Zaki SA, Damiati SA, Rijal HB, Hagishima A, AbdRazak A (2017). Adaptive thermal comfort in university classrooms in Malaysia and Japan. Build Environ.

[CR85] Zaki SA, Rosli MF, Rijal HB, Sadzli FNH, Hagishima A, Yakub F (2021) Effectiveness of a cool bed linen for thermal comfort and sleep quality in air-conditioned bedroom under hot-humid climate. Sustainability 13(16):9099. 10.3390/su13169099

[CR86] Zheng P, Wang C, Liu Y, Lin B, Wu H, Huang Y, Zhou X (2022). Thermal adaptive behavior and thermal comfort for occupants in multi-person offices with air-conditioning systems. Build Environ.

