# Supplementary material for: Assessment of indoor thermal comfort temperature and related behavioural adaptations: a systematic review
Source: Environ Sci Pollut Res Int. 2023 May 22;30(29):73137–49. doi: 10.1007/s11356-023-27089-9 (PMC10287772; doi:10.1007/s11356-023-27089-9)
Supplement: Supplementary file 1 — Supplementary file1 (DOCX 21 KB) [file 11356_2023_27089_MOESM1_ESM.docx]

**Table S1**. Navigation Guide Systematic Review Ratings of Each Study

| **No** | **Study** | **Recruitment** | **Blinding** | **Exposure Assessment** | **Outcome Assessment** | **Confounding** | **Incomplete Outcome Data** | **Selective Reporting** | **Conflicts Of Interest** | **Other Bias** | **Overall**  **Risk Of Bias** |
| --- | --- | --- | --- | --- | --- | --- | --- | --- | --- | --- | --- |
|  | (Yao et al. 2010) | Low | Low | Low | Low | Low | Low | Low | Low | Low | Low |
|  | (Teli et al. 2012) | Low | Low | Low | Low | Low | Low | Low | Low | Low | Low |
|  | (Pellegrino et al. 2012) | Low | Low | Low | Low | Low | Probably Low | Low | Low | Low | Low |
|  | (Liang et al. 2012) | Low | Low | Probably Low | Low | Low | Low | Low | Low | Low | Low |
|  | (Rijal 2014) | Low | Low | Low | Low | Low | Low | Low | Low | Low | Low |
|  | (Baruah et al. 2014) | Probably Low | Low | Probably Low | Low | Probably Low | Probably Low | Low | Low | Low | Probably Low |
|  | (Yun et al. 2014) | Low | Low | Low | Low | Low | Low | Probably Low | Low | Low | Low |
|  | (De Dear et al. 2015) | Low | Low | Low | Low | Low | Low | Low | Low | Low | Low |
|  | (Singh 2016) | Low | Low | Low | Low | Low | Low | Low | Low | Low | Low |
|  | (Damiati et al. 2016) | Low | Low | Low | Low | Probably Low | Low | Low | Low | Low | Low |
|  | (Wang et al. 2017) | Probably Low | Low | Low | Low | Probably Low | Low | Low | Low | Low | Low |
|  | (Liu et al. 2017) | Low | Low | Probably Low | Low | Probably Low | Low | Low | Low | Low | Low |
|  | (Haddad et al. 2017) | Low | Low | Low | Low | Probably Low | Low | Low | Low | Low | Low |
|  | (Zaki et al. 2017) | Low | Low | High | Low | Probably Low | Probably Low | Low | Low | Low | Low |
|  | (Liu & Yong Yau. 2017) | Low | Low | Low | Low | Low | Low | Low | Low | Low | Low |
|  | (Singh et al. 2018) | Low | Low | Probably Low | Low | Probably Low | Probably Low | Low | Low | Low | Probably Low |
|  | (Khalid et al. 2019) | Low | Low | Low | Low | Low | Probably Low | Low | Low | Low | Low |
|  | (Hossain et al. 2019) | Low | Low | Low | Low | Low | Probably Low | Probably Low | Low | Low | Low |
|  | (Kumar & Singh 2019) | Low | Low | Low | Low | Probably Low | Probably Low | Probably Low | Low | Low | Probably Low |
|  | (Rijal 2019) | Low | Low | Low | Low | Low | Low | Low | Low | Low | Low |
|  | (Kim et al. 2019) | Low | Low | Low | Low | Low | Probably Low | Low | Low | Low | Low |
|  | (Wu et al. 2019) | Low | Low | Low | Low | Low | Low | Low | Low | Low | Low |
|  | (Korsavi & Montazami 2020) | Low | Low | Probably Low | Low | Probably Low | Probably Low | Low | Low | Low | Probably Low |
|  | (Malik et al. 2020) | Low | Low | Low | Low | Low | Low | Low | Low | Low | Low |
|  | (Budiawan & Tsuzuki 2021) | Low | Low | Probably Low | Low | Probably Low | Low | Low | Low | Low | Low |
|  | (Shrestha et al. 2021) | Low | Low | Probably Low | Low | Low | Low | Low | Low | Low | Low |
|  | (Draganova et al. 2021) | Low | Low | Low | Low | Probably Low | Probably Low | Low | Low | Low | Low |
|  | (Tsuzuki et al. 2021) | Low | Low | Probably Low | Low | Low | Low | Low | Low | Low | Low |
|  | (Zaki et al. 2021) | Low | Low | Low | Low | Probably Low | Low | Low | Low | Low | Low |
|  | (Sun et al. 2022) | Low | Low | Low | Low | Low | Low | Low | Low | Low | Low |
|  | (Zheng et al. 2022) | Low | Low | Low | Low | Probably Low | Low | Low | Low | Low | Low |
